# *Phaseolus coccineus* Seed: A Valued Resource for Bioactive Compounds Targeting Health and Tumor Cells

**DOI:** 10.3390/ijms26052189

**Published:** 2025-02-28

**Authors:** Rita Russo, Antonio Colantuono, Sonia Di Gaetano, Domenica Capasso, Annalisa Tito, Emilia Pedone, Luciano Pirone

**Affiliations:** 1 Institute of Biostructures and Bioimaging, Via Pietro Castellino 111, 80131 Naples, Italy; rita.russo@unicampania.it (R.R.); sonia.digaetano@cnr.it (S.D.G.); 2 Department of Environmental, Biological and Pharmaceutical Sciences and Technologies, University of Campania “Luigi Vanvitelli”, 81100 Caserta, Italy; 3 Arterra Bioscience S.p.A., Via Benedetto Brin 69, 80142 Napoli, Italy; antonio@arterrabio.it (A.C.); annalisa@arterrabio.it (A.T.); 4 Department of Physics “Ettore Pancini”, University of Naples Federico II, Via Cinthia 4, 80126 Naples, Italy; domenica.capasso@unina.it

**Keywords:** galectin, Gal-3, *Phaseolus coccineus* seed, galectins inhibitors

## Abstract

Human galectin-3 (Gal-3), a β-galactoside binding lectin through its Carbohydrate Recognition Domain (CRD), is implicated in a wide range of cellular functions and is involved in critical biological processes including pathogen recognition, immune response, inflammation and fibrosis. Recently, Gal-3 has gained increasing attention for its role in pathological conditions such as cancer, where it influences cancer growth and progression, inflammatory processes and oxidative stress, opening the search for potential inhibitors. In this context, several naturally derived molecules have attracted particular interest, some of them being used in clinical trials. Here, we used the seeds of the legume *Phaseolus coccineus* as a green resource for bioactive compounds. The peptide-rich crude extracts were chemically characterized for their peptide and polyphenol contents, as well as their in vitro antioxidant activity, and the powerful obtained extract was tested for biological activities such as cytotoxicity and antioxidant and anti-inflammatory effects on cellular models. Furthermore, the interaction between the crude extract and the CRD of recombinant Gal-3 was verified with the aim of associating its biological effects with the inhibition of Gal-3 activity.

## 1. Introduction

Human galectin-3 (Gal-3), a β-galactoside-binding lectin, has been discovered to be a unique chimera-type galectin among the sixteen members of the galectin family, displaying peculiar structural features such as the N-terminal non-lectin domain involved in its pentamerisation and the NGWR motif in its Carbohydrate Recognition Domain (CRD) [1]. It is considered a multifaced protein due to its involvement in a wide range of cellular interactions, and it is required as a key regulator of critical biological processes, including pathogen recognition, immune response, inflammation and fibrosis [2,3,4].

As a biomarker and potential therapeutic target, Gal-3 is gaining increasing attention for its role in pathological conditions such as neurodegenerative disorders and cancer [5,6,7].

In this context, Gal-3 plays a prominent role in the tumour microenvironment, where it is mostly overexpressed, influencing not only cancer growth and progression but also inflammatory processes and oxidative stress, opening up the search for potential inhibitors [8,9,10,11]. Thus, since most of the activities of Gal-3 are associated with its carbohydrate-binding characteristics, the inhibition of the CRD by synthetic antagonists able to compete with the natural ligand seems to be a feasible option for therapeutic intervention; indeed, several inhibitors have been developed, and some of them are currently being evaluated in phase I or phase II clinical trials for various cancer types [12,13]. Additionally, another promising strategy in the search for potential Gal-3 inhibitors has been to extract large molecules from natural sources that could not only mask the CRD-binding groove but also create engulfment in other portions of the protein that have recently been shown to be essential for recognition specificity and increased binding affinity. This strategy has led to the production of several naturally derived molecules, some of which have been used for clinical trials [14,15,16,17].

On this basis, we used the seeds of the leguminous plant *Phaseolus coccineus* as a green resource for bioactive compounds, and we developed and standardised extraction protocols for peptide-rich crude extracts. *Phaseolus coccineus* is a pulse with a high content of protein, and it is rich in phytochemicals that possess activities beneficial to health. The potential applications of the extract in different fields are numerous, yet there have been few studies reporting a complete characterization of the bioactive compounds present in species such as *P. coccineus*, *P. lunatus*, and *P. polyanthus* [18,19]. Here, we present the chemical characterization of seeds of the legume *Phaseolus coccineus* in terms of their peptide and polyphenol contents, as well as their in vitro antioxidant activity. Additionally, the powerful obtained extract, denominated AsP100, was biologically evaluated for its cytotoxicity, antioxidant effects, and anti-inflammatory effects on a cellular model. The interactions between the crude AsP100 extract and the CRD of recombinant Gal-3 were observed with the aim of associating its biological effects with the inhibition of Gal-3 activity.

## 2. Results and Discussion

### 2.1. Extract Preparation

The seeds of *Phaseolus coccineus* were washed, dried at −80° C and homogenized. The obtained powder was resuspend in water and homogenised. The obtained suspension was subjected to different enzymatic treatments and a heat treatment. For each treatment, a specific control was also produced. In total, eight samples were obtained:

1. PaP—it was treated with Viscozyme^R^ + Papain;
2. CtrlPaP—it underwent the same processes as PaP (1) without the addition of any enzyme;
3. PaP100—it was treated with Viscozyme^R^ + Papain and subsequently boiled;
4. CtrlPaP100—it underwent the same processes as PaP100 (3) without the addition of any enzyme;
5. AsP—it was treated with ViscozymeR + Aspergillopepsin I;
6. CtrlAsP—it underwent the same processes as AsP (5) without the addition of any enzyme;
7. AsP100—it was treated with ViscozymeR + Aspergillopepsin I and subsequently boiled;
8. CtrlAsP100—it underwent the same processes as AsP100 (7) without the addition of any enzyme.

Every sample derived from the same preparation, with the samples divided equally at every step; thus, any biases in the procedure were avoided.

### 2.2. Peptide Content

**Figure 1:**
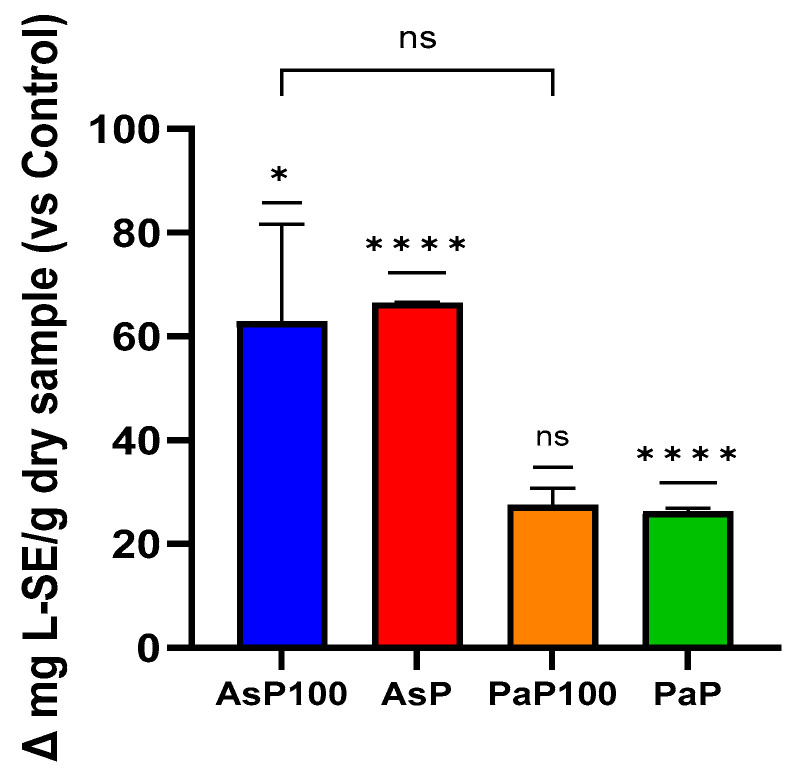
O-phthalaldehyde assay for peptide content. Comparative analysis between the AsP100 and AsP extracts (respectively treated with Aspergillopepsin I + heat and Aspergillopepsin I only) and the PaP100 and PaP extracts (respectively treated with Papain + heat and Papain only); the peptide amount of each sample is expressed as Δ mg of L-Serine equivalent compared with respective controls. Statistical significance was determined with an unpaired *t*-test. * *p*-value < 0.05, **** *p*-value < 0.0001, ns = not significant.

Firstly, to validate the efficacy of the enzymatic treatment with Aspergillopepsin I (in AsP and AsP100 extracts) and Papain (in PaP100), we performed an OPA assay, which allowed us to evaluate the increasing peptide content in the enzymatically treated extracts and compare the activity of the selected enzymes. These experiments confirmed that the enzymatic treatment led to an increased peptide content in all extracts (Figure 1). Surprisingly, the comparison between the enzyme- and heat-treated mixtures (AsP100 and PaP100) with the enzyme-only-treated mixtures (AsP and PaP) showed that heat treatment, which is important for enzyme inactivation, was not responsible for the loss of peptides. Between the two double-treated extracts, AsP100 was richer in peptides compared with PaP100.

### 2.3. Total Polyphenol Content and In Vitro Antioxidant Activity

**Figure 2:**
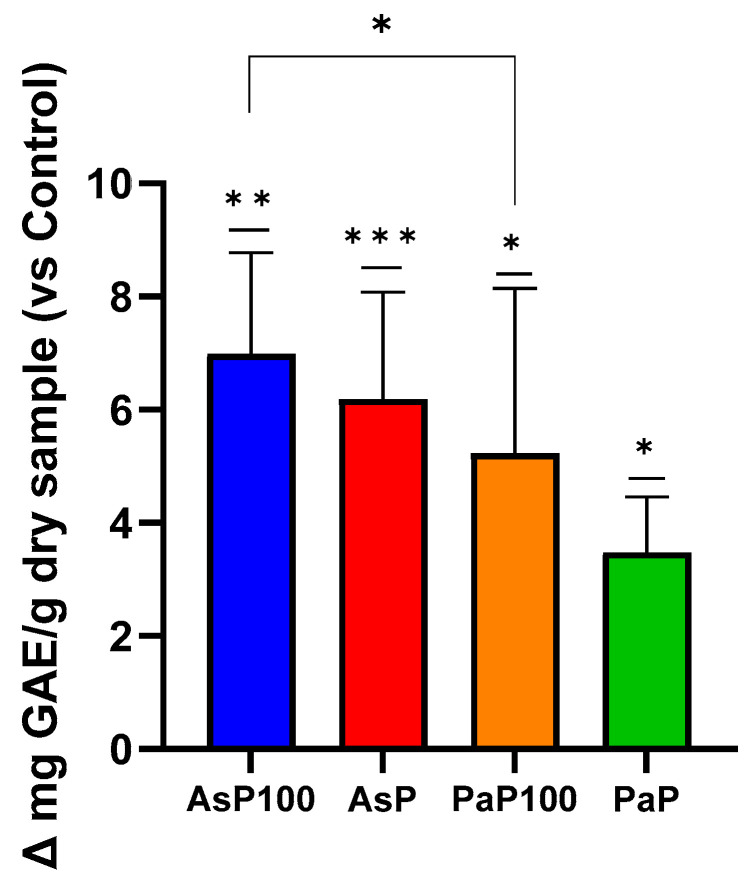
Folin–Ciocalteu assay for total polyphenol content. Comparative analysis between the AsP100 and AsP extracts (respectively treated with Aspergillopepsin I + heat and Aspergillopepsin I only) and the PaP100 and PaP extracts (respectively treated with Papain + heat and Papain only); the results of each sample are expressed as Δ mg of L-Serine equivalent/g of dry sample compared with respective controls. Statistical significance was determined with an unpaired *t*-test. * *p*-value < 0.05, ** *p*-value < 0.01, *** *p*-value < 0.001,.

The total polyphenol content was determined with a Folin–Ciocalteu assay. Comparing the GAE value obtained for each sample with its respective control, the experiment showed that all enzymatically treated extracts, in combination or not with the heat treatment, had higher polyphenol contents than their relative controls (Figure 2). Comparing the AsP100 sample with AsP, it could be observed that the combination of enzymatic and heat treatments produced the highest amount of polyphenols in the extraction mixture. The same was true for the PaaP100 and PaP samples. Additionally, between the two double-treated extracts, AsP100 was a bit more abundant in polyphenols compared with PaP100.

**Figure 3:**
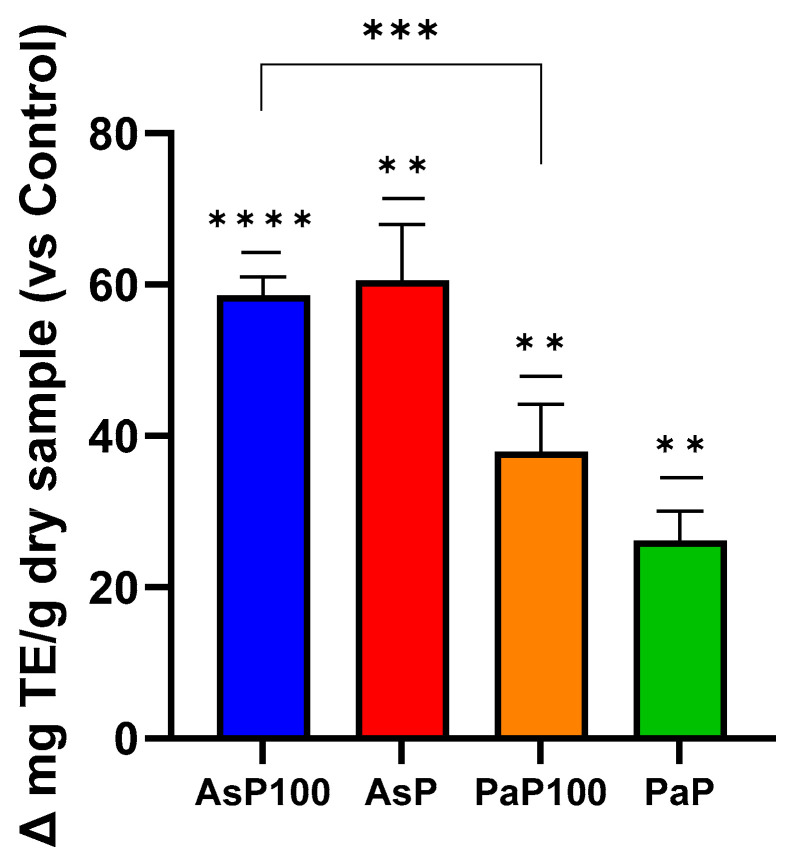
ABTS assay for in vitro antioxidant activity. Comparative analysis between the AsP100 and AsP extracts (respectively treated with Aspergillopepsin I + heat and Aspergillopepsin I only) and the PaP100 and PaP extracts (respectively treated with Papain + heat and Papain only); the results of each sample are expressed as Δ mg of Trolox equivalent/g of dry sample compared with respective controls. Statistical significance was determined with an unpaired *t*-test. ** *p*-value < 0.01, *** *p*-value < 0.001, **** *p*-value < 0.0001.

Next, an ABTS assay was performed to quantify the free radical scavenging capacity of a sample, which correlates with its protective potential against oxidative stress. This capacity is also often related to the polyphenolic composition of a sample. This experiment highlighted that AsP and AsP100 had the stronger in vitro antioxidant activity; between them, the AsP extract was the most antioxidant (Figure 3). However, considering the requirement of a heat treatment to inactivate enzymes, AsP100 could be considered the most antioxidant extract.

### 2.4. Evaluation of Antiproliferative Activity

**Figure 4:**
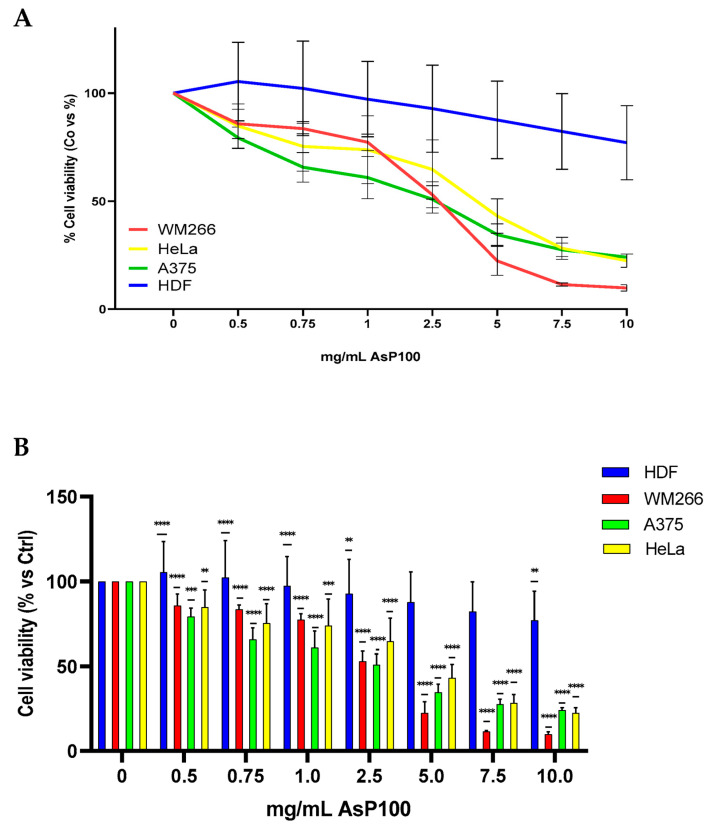
MTT assay for cell viability evaluation. First, 2 × 10^3^ WM266/HDF and 1.2 × 10^3^ A375/HeLa/well were seeded; after 24 h, they were treated with increasing concentrations of the AsP100 extract. After 48 h, the percentages of live cells were determined with an MTT assay. (**A**) Using the online software https://www.aatbio.com/tools/ic50-calculator (accessed on 1 September 2023) [20], the IC_50_ values of the selected cell lines were calculated: WM266 = 2.46 mg/mL; A375 = 2.0 mg/mL; HeLa = 3.45 mg/mL; HDF = 37.19 mg/mL. The chosen calculator option was the three-parameter mode; data were analysed using the equation described in Section 3.5. (**B**) Bar plot with median ± SD. Statistical significance was determined with a paired *t*-test. ** *p*-value < 0.01, *** *p*-value < 0.001, **** *p*-value < 0.0001.

The most promising obtained extract, AsP100, was further used to analyse its effect on cell vitality and proliferation on different in vitro human cell lines. The cytotoxic effect was tested with an MTT assay on different cell lines: human dermal fibroblasts (HDFs), two different human metastatic melanoma cell lines (A375 and WM266), and human cervical carcinoma (HeLa). The results revealed an interesting differential cytotoxicity on the tumour lines compared with the health line, with a stronger antiproliferative potential on tumour cells compared with the healthy one (Figure 4). The IC_50_ values, calculated by using the online software https://www.aatbio.com/tools/ic50-calculator (accessed on 1 September 2023) [20], are reported in the caption below.

These results demonstrated that AsP100 possesses significant specific antiproliferative activity on tumour cells, particularly on the two metastatic lines (WM266 and A375), as distinguished by its limited toxicity on healthy cells. The results are comparable with other data in the literature reporting a similar effect using analogous concentration ranges [21]. This selectivity indicates that it may be used as a potential safe and effective therapeutic support, preferentially targeting neoplastic cells.

### 2.5. IL-6 Released Evaluation

**Figure 5:**
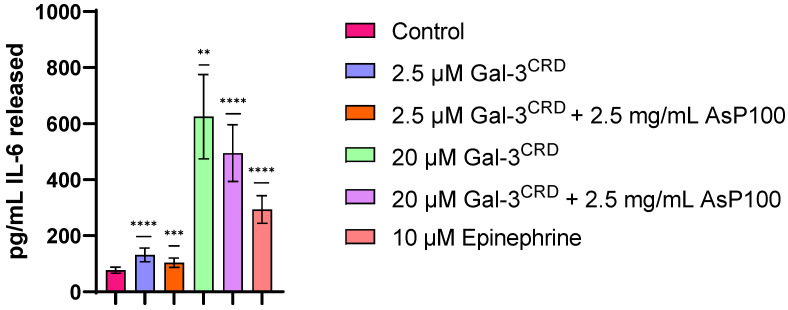
ELISA for IL-6 detection. 1 × 10^4^ HDF/well were seeded in a 96-well plate. After 24 h, HDFs were treated with two concentrations of recombinant Gal-3^CRD^ in the presence and absence of 2.5 mg/mL of AsP100. The positive control is represented by 10 μM of epinephrine. After 24 h, the HDF supernatant was collected and used to quantify the IL-6 released in the media using a Human ELISA IL-6 kit. The data are reported as pg/mL of IL-6 released in the media. Statistical significance was determined with a paired *t*-test. ** *p*-value < 0.01, *** *p*-value < 0.001, **** *p*-value < 0.0001.

Considering that Gal-3 has been demonstrated to be a pro-inflammatory molecule in several contexts, we researched the potential anti-inflammatory effects of the AsP100 extract alone and in co-treatment with recombinant Gal-3^CRD^, using the recombinant protein as inflammation inductor. Briefly, 1 × 10^4^ HDF cells were treated for 48 h with recombinant Gal-3^CRD^ in the presence and absence of AsP100 to investigate both Gal-3^CRD^’s inflammatory role and the potential anti-inflammatory effect of the extract. Epinephrine was used as a positive control due to its well-known role in stimulating IL-6 release. For each treatment, IL-6 detection was performed by analysing the conditioned media using the Human IL-6 ELISA kit (Figure 5). The treatments with 2.5 μM and 20 μM of Gal-3^CRD^ confirmed a dose-dependent pro-inflammatory effect on HDF cells; co-treatment with 2.5 mg/mL of the AsP100 extract resulted in slight reductions in IL-6 to 26.4% and 26.9%, respectively.

### 2.6. BLI Interaction Analysis

**Figure 6:**
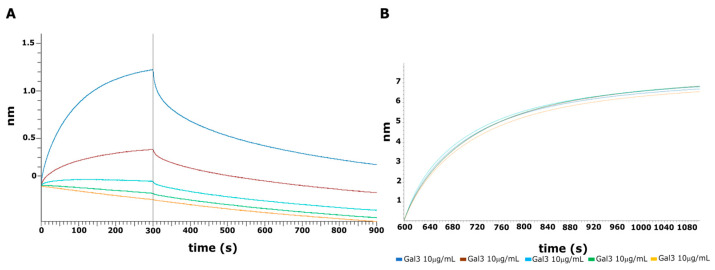
BLI interaction analysis. (**A**) Association and dissociation curves between Gal-3^CRD^ and AsP100 in a range of concentrations from 10 mg/mL to 0.3 mg/mL. In detail: blue, brown, cyan, green and orange lines correspond to 10 mg/mL, 5 mg/mL, 2.5 mg/mL, 1 mg/mL, and 0.3 mg/mL, respectively. (**B**) Loading curves of Gal-3^CRD^ immobilized on Ni^+^-NTA biosensors at 10 μg/mL.

To confirm the effect of the crude AsP100 extract on Gal-3^CRD^, we performed an interaction assay using bio-layer interferometry (BLI) technology. Thus, 10 μg/mL of recombinant protein was immobilized on Ni^+^-NTA Octet^R^ biosensors via polyHis-tag. The AsP100 extract was tested in a range of concentrations from 0.3 mg/mL to 10 mg/mL (Figure 6A). A negative control was made by immobilizing a dumb protein POZ1 and testing it versus the maximum concentration of AsP100 used (Figure 6B). The negative control showed a certain binding level, an expected result given the complex and non-specific environment of the extract, which generated background noise. The results clearly demonstrated the presence of bio-compounds in the crude AsP100 extract that interact with Gal-3.

### 2.7. ROS Assay

**Figure 7:**
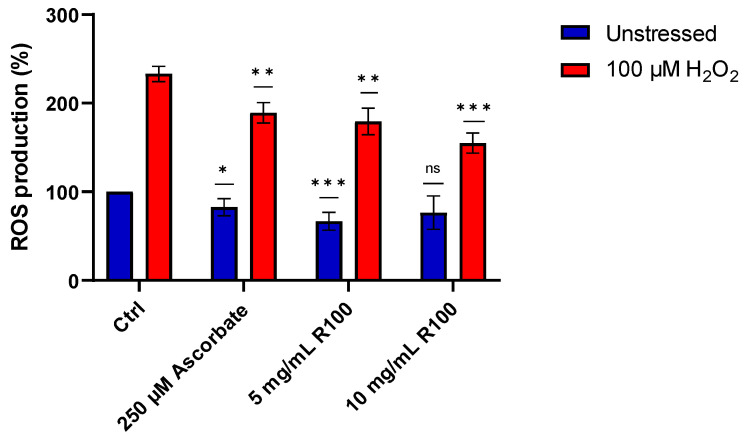
Percentages of ROS production on HDF cells treated with ascorbate (positive control) and different concentrations of the AsP100 extract. Statistical significance was determined with a paired *t*-test. * *p*-value < 0.05, ** *p*-value < 0.01, *** *p*-value < 0.001, ns = not significant.

After demonstrating the in vitro antioxidant activity of the AsP100 extract, we performed an ROS assay to evaluate the existing correspondence of its antioxidant properties on a cellular model. Thus, HDF cells were treated for 24 h with two concentrations of the AsP100 extract (5 and 10 mg/mL). Successively, cells were treated with 100 μM H_2_O_2_ to induce ROS production; in this way, it was possible to investigate the role of AsP100 in the presence of both physiological and high concentrations of ROS species. Ascorbate, a well-known antioxidant molecule with strong activity on ROS scavenging, was used as a positive antioxidant control. The HDF results confirmed the observations made in vitro, highlighting a dose-dependent antioxidant activity, similar to ascorbate (Figure 7).

## 3. Materials and Methods

### 3.1. Extract Preparation

The seeds of *Phaseolus coccineus* were acquired from La Semiorto Sementi s.r.l. (Sarno, Italy). The seeds were washed in citric acid 3% (1/10, *w*/*v*) for 30 min; the citric acid was removed by washing with sterile H_2_O two times. Next, the seeds were dried and frozen at −80 °C. To obtain a powder, the seeds were homogenized 3 min at 1500 rpm and 1 min at 3800 rpm using a Grindomix GM 300 knife mill (Retsch GmbH, Haan, Germany). The obtained powder was resuspended in sterile H_2_O (1/5, *w*/*v*); the obtained suspension was homogenized 6 min at 3800 rpm. The pH of the suspension was adjusted to 4.2 with 12 N HCl for subsequent enzymatic treatment. To digest complex carbohydrates, the suspension was treated with Viscozyme^R^ (Sigma-Adrich, St. Louis, MO, USA), a broad-spectrum enzyme used to hydrolyse plant tissue, with 1/1000 *v*/*v* of H_2_O. The treatment was performed for 4 h at 37 °C under stirring at 150 rpm. Next, the mixture was divided into two equal parts and separately treated with two different enzymes. 1: AsP100 was obtained by treating with 4 g/kg of starting material of Aspergillopepsin I (CAS Number 9025-49-4, Sigma, St. Louis, MO, USA), a hydrolytic enzyme that cuts between hydrophobic residues; 2: the so-called PaP100 was produced with a treatment of 4 g/kg of a Papain starting material (CAS Number 9001-73-4, Sigma, St. Louis, MO, USA) that specifically cuts near cysteine residues. The optimum pH values for Aspergillopepsin I and Papain enzymes are, respectively, 2.5 and 7.0, so the pH values of the two suspensions were separately adjusted with 12 N HCl and 10 M NaOH. For both, the treatment was performed over night at 50 °C under stirring at 150 rpm. Subsequently, the double-digested suspensions were centrifuged for 20 min at 6300 rpm and 4 °C, and the supernatant was filtered through filter paper (FILTER-LAB, Barcelona, Spain). To inactivate the hydrolytic enzymes used during the process, the obtained suspensions were boiled for 5 min at 100 °C under stirring at 150 rpm. To understand the effect of boiling, we prepared additional samples that underwent the double enzymatic treatment but were not exposed to heat (AsP and PaP, respectively treated with Viscozyme^R^ + Aspergillopepsin I and Viscozyme^R^ + Papain). Then, suspensions were centrifuged for 10 min at 6300 rpm and 4 °C and filtered as before. Ultimately, the pH of both extracts was adjusted to 7.0 with 10 N NaOH and then freeze-dried until we obtained a fine powder. The controls (CtrlAsP, CtrlAsP100, CtrlPaP and CtrlPaP100) were prepared the same as the relative samples without the addition of any enzymatic treatments. All the samples and controls were freshly prepared as 5% stock solutions solubilizing the powder in sterile H_2_O, centrifuged for 10 min at 4500 rpm and 4 °C, and filtered with a 0.45 µm syringe filter (Euroclone, Pero, MI, Italy).

### 3.2. Peptide Content

An OPA assay was used to estimate the amount of total peptides in order to check the success of the enzymatic treatment [22]. It was performed in a 12-well plate. The OPA solution was freshly made by diluting and combining 0.05 M sodium tetraborate, SDS and OPA. For the standard curve, an appropriately diluted L-Serine solution was used. Each sample (AsP, AsP100, PaP, PaP100 and relative controls) was rightly diluted in 2 mL of OPA reagent and incubated for 2 min in the dark, and the absorbance was detected at 340 nm using a Victor Nivo multiplate reader (Perkin Elmer, Woodbridge, ON, Canada). Results are expressed as mg L-Ser equivalent (L-SE)/g of dry sample. This assay was conducted in triplicate and repeated at least three times.

### 3.3. Total Polyphenol Content

The amount of total phenols was spectrophotometrically assessed using a Folin–Ciocalteu assay, as described by [23]. Briefly, a reaction between 125 µL of each appropriately diluted sample (AsP, AsP100, PaP, PaP100 and relative controls) and 125 uL of a Folin–Ciocalteu phenol reagent was carried out for 6 min. Next, 1.25 mL of a 7.5% Na_2_CO_3_ solution was added and allowed to react for 90 min in the dark. The absorbance at 760 nm was measured. The results are expressed as mg of gallic acid equivalents/g of dry sample. This assay was conducted in triplicate and repeated at least three times.

### 3.4. In Vitro Antioxidant Activity

The antioxidant capacity of the samples (AsP, AsP100, PaP, PaP100 and relative controls) was measured by using an ABTS assay, as reported by [24]. Briefly, a stable stock solution of ABTS + was produced by reacting a 7 mmol/L aqueous solution of ABTS with 2.45 mmol/L of potassium persulfate (final concentration) and allowing the mixture to stand in the dark at 4 °C for 16 h before use. The ABTS + solution was diluted with PBS to an absorbance of 0.700 ± 0.05 at 734 nm. Each sample was appropriately diluted in H_2_O, and 0.1 mL of diluted solution was added to 1 mL of the ABTS·+ solution. The mixture was allowed to stand at room temperature for 2.5 min before the absorbance was recorded at 734 nm by using a multiplate reader (EnSpire, Perkin Elmer, Waltham, MA, USA). The results are expressed as μmol Trolox equivalents (TE)/g of dry sample. This assay was conducted in triplicate and repeated at least three times.

### 3.5. Cell Lines and Culture Condition

The human metastatic melanoma (WM266) cell line was a kind gift of Dr. Carla Maria Rozzo (Istituto di Genetica e Biomedica, CNR, Sassari); normal human dermal fibroblasts (HDFs) were acquired from Life Technology (Invitrogen, Waltham, MA, USA); the human adenocarcinoma cell line (HeLa) and human low metastatic melanoma (A375) were from ATCC (Manassas, VA, USA). HDFs, HeLa and A375 were grown in DMEM supplemented with 10% FBS, 1% L-Glutamine, 100 U/mL of penicillin, and 100 µg/mL streptomycin (Euroclone, MI, Italy). Under the same experimental conditions, WM266 cell lines were grown in RPMI supplemented with heat-inactivated 10% FBS, 2.5 mM of glutamine, 100 U/mL of penicillin, and 100 µg/mL of streptomycin (Euroclone, MI, Italy). All cell lines were maintained in humidified air containing 5% CO_2_ at 37 °C.

### 3.6. Cell Proliferation Assay

To assess the effect of the AsP100 extract on the viability of healthy and tumoral cells, a cytotoxicity assay was performed. We investigated the effects of the AsP100 extract on the proliferation of normal human dermal fibroblasts (HDF), two different metastatic melanoma cell lines (A375, WM266), and a cervical carcinoma cell line (HeLa). At a density of 2.0 × 10^3^ (HDF, WM266) or 1.2 × 10^3^ (A375, HeLa) cell/well, all cell lines were seeded in a 96-well plate. After 24 h of seeding, cells were treated with increasing concentrations of the AsP100 extract for 48 h. Cell proliferation was determined with an MTT assay (Sigma-Adrich, St. Louis, MO, USA) [25]. Briefly, after 48 h of treatment, the media were removed and replaced with 100 µL of 500 µg/mL MTT in each well (the stock solution was 5 mg/mL in culture medium without phenol red). After 4 h of incubation in the dark, the MTT solution was removed and 100 µL of dissolving solution (HCl, Triton 100X, 2-propanol) was put in. After 30 s of shaking, the absorbance at 570 nm was measured with a 2300 multi-mode microplate reader (EnSpire, Perkin Elmer, Waltham, MA, USA). The mean value of proliferating cells for each treatment was compared with untreated cells (control). The IC_50_ values were calculated using Quest Graph^TM^ IC_50_ Calculator provided by https://www.aatbio.com/tools/ic50-calculator (accessed on 1 September 2023). The chosen calculator option was the three-parameter mode; data were analysed by using the following equation:

$$Y=\mathrm{Min}+\frac{\mathrm{Max}-\mathrm{Min}}{\;\;\;\;\;\;1+{\left(\frac{X}{\mathrm{IC}50}\right)}^{\mathrm{Hill}\;\mathrm{coefficient}}}$$

### 3.7. ELISA for IL-6 Detection

To evaluate the potential anti-inflammatory effect of AsP100 treatment on healthy cells, an IL-6 ELISA Kit (Abcam 178013, Cambridge, UK) was used to detect the level of pro-inflammatory IL-6 release from cells. Firstly, the pro-inflammatory effect of recombinant Gal-3^CRD^ was assessed by treating cells with increasing concentrations of the protein. Once this role was demonstrated on HDF cells, it was used at its maximum tested concentration (20 μM) to evaluate the potential anti-inflammatory role of the AsP100 extract. Thus, 1 × 10^4^ human dermal fibroblasts (HDFs) were seeded in a 96-well plate. After 24 h, the cells were treated with increasing concentrations of the AsP100 extract and a combination of AsP100 with Gal-3^CRD^. A solution of 10 µM epinephrine was used as a positive control since it is a well-known IL-6 release inductor. After 24 h, the conditioned media were transferred to a 96-well immuno-plate provided by the kit and incubated for 1 h at RT with the Ab cocktail. After a wash step, 100 μL/well of the TMP development solution was added and left reacting for 10 min in the dark. The development reaction stopped following the addition of a 100 μL/well Stop solution. The absorbance at 450 nm was measured using a 2300 multi-mode microplate reader (EnSpire, Perkin Elmer, Waltham, MA, USA). The human IL-6 protein was serially diluted to obtain the standard curve necessary for data analysis. The results are the mean value ± SE of at least three independent experiments conducted in triplicate.

### 3.8. ROS Detection

The antioxidant activity of a cellular model is assessable with an ROS assay [26]. It is based on the transformation of CM-DCFDA dye, in the presence of high concentrations of O_2_ reactive species, into the fluorescent product DCF. Briefly, 4 × 10^3^ human dermis fibroblasts (HDFs) were seeded in a 96-well plate. After 24 h, cells were incubated for 2 h with different concentrations of the AsP100 extract (1.25, 2.5 and 5 mg/mL), and a positive control was treated with 250 μM of ascorbate for the same time. After incubation, cells were washed in PBS and incubated with the CM-DCFDA (Invitrogen, Waltham, MA, USA) for 30 min at 37 °C, following the manufacturer’s instructions. Next, cells were washed in PBS, and half of the samples were treated for 30 min with 100 μM of H_2_O_2_ to induce ROS formation. The fluorescence intensity was measured at 353 nm (excitation at 490 nm) using a 2300 multi-mode microplate reader (EnSpire, Perkin Elmer, Waltham, MA, USA). Data were analysed by using GraphPad Prism v. 9.5. The results are the mean value ± SE of at least three independent experiments conducted in triplicate.

### 3.9. Protein Expression and Purification

The genes encoding the CRD of human galectin-3 (Gal-3^CRD^, residues 112–250) were purchased from Genewiz (South Plainfield, NJ, USA). The recombinant protein was expressed and purified as described elsewhere [11]. For BLI and cellular experiments, Gal-3^CRD^ was exchanged in PBS, 1% DMSO.

### 3.10. BLI Interaction Analysis

To analyse if there were bioactive compounds inside the ASP100 extract that interacted with Gal-3^CRD^, an interaction analysis using bilayer interferometry (BLI) was performed. The protein was appropriately diluted in a kinetic buffer (PBS containing 0.02% Tween20, 0.1% BSA, and 0.05% NaN_3_) and successfully immobilized at 10 µg/mL on an Octet^R^ Ni^2+^-NTA sensor chip (Sartorius, Gottinga, Germany) according to the manufacturer’s instructions. A dumb protein polyHis-tag POZ1, the main domain of the KCTD protein family involved in the modulation of ion channels [27], was immobilized at 10 µg/mL and used as a control to estimate the unspecific interactions. The ASP100 extract was appropriately diluted in the kinetic buffer at increasing concentrations (from 10 mg/mL to 0.313 mg/mL, serially diluted 1:2) and tested for binding at 25 °C. All mathematical manipulations were carried out using Octet Analysis Studio 12.2 software. The best fit for the experimental curves was obtained by selecting the heterogeneous model provided by the Octet Analysis software.

### 3.11. Statistical Data Analysis

All data are presented as mean values ± SE. The statistical analysis was performed using ordinary paired or unpaired Student’s *t*-tests, and *p* < 0.05 was considered significant.

## 4. Conclusions

Here, we performed a chemical characterisation of the seeds of the legume Phaseolus coccineus in terms of their peptide and polyphenol contents. Our focus was on a peptide-rich extract subjected to enzymatic and thermal treatments. We report the evaluation of the biological roles of a new *Phaseolus coccineous* peptide-rich extract named AsP100. This crude extract, characterized by its polyphenol and peptide contents and by its in vitro antioxidant potential, showed interesting biological activities on different cellular models. First, it had a strong antiproliferative effect on selected tumour cells accompanied by a safe profile on the dermal fibroblasts used as a healthy model. In addition, we also investigated the impact of AsP100 treatments on ROS production by HDF cells, observing an antioxidant profile. Moreover, we found that AsP100 may be also considered for its anti-inflammatory potential due to its negative influence on the IL-6 release of HDF cells. In conclusion, AsP100 represents a complex, naturally derived new matrix that could gain attention for its roles in cell metabolism as a multifaceted beneficial supplement. Considering the positive results obtained in binding analysis, we can hypothesize that the mechanisms whereby it acts could involve Gal-3.

Although further experiments need to be carried out on a purified extract of AsP100 to verify the uniqueness of its biological effects and interaction with Gal-3, a possible application of this extract on tumour cells could be hypothesised, taking advantage of the collected data demonstrating its low impact in terms of potential side effects.

## Data Availability

Data is contained within the article.

## Abbreviations

Gal-3	Galectin-3
CRD	Carbohydrate Recognition Domain
OPA	o-phthalaldehyde
HDF	Human dermal fibroblast
HeLa	Human cervical carcinoma
